# C‐reactive protein predicts the development of walled‐off necrosis in patients with severe acute pancreatitis

**DOI:** 10.1002/jgh3.12605

**Published:** 2021-06-29

**Authors:** Junichi Fujiwara, Satohiro Matsumoto, Masanari Sekine, Hirosato Mashima

**Affiliations:** ^1^ Department of Gastroenterology, Saitama Medical Center Jichi Medical University Saitama‐shi Saitama Japan

**Keywords:** C‐reactive protein, severe acute pancreatitis, walled‐off necrosis

## Abstract

**Background and Aim:**

Walled‐off necrosis (WON) is reported to occur in 1–9% of patients with acute pancreatitis. However, the factors associated with the onset of this condition have not been elucidated. This study aimed to investigate the potential predictive factors for WON in patients diagnosed with severe acute pancreatitis at our hospital.

**Methods:**

This study included 26 patients with severe acute pancreatitis identified among the 211 patients with acute pancreatitis admitted to our hospital between January 2014 and December 2018. Patients with and without WON (WON and non‐WON groups, respectively) were compared to identify potential factors involved in the onset of this condition.

**Results:**

The 26 patients had a median age of 67 years, and 65% were male. WON occurred in 15 patients (57.7%). In a univariate analysis, the WON and non‐WON groups differed significantly in terms of maximum C‐reactive protein (CRP) levels (median) (322.7 mg/L vs 163.8 mg/L [*P* = 0.001]). In a multivariate analysis, a significant association was identified between the maximum CRP level and the onset of WON (odds ratio: 1.20, 95% confidence interval: 1.05–1.37). The CRP level peaked within 3 days in 88%.

**Conclusion:**

The maximum CRP level was identified as a predictive factor for the onset of WON, and a high proportion of patients with WON exhibited elevated CRP levels within 3 days after diagnosis. This work suggests the clinical importance of continuous monitoring at an early stage after diagnosis to identify the maximum CRP level.

## Graphical abstract

The maximum C‐reactive protein (CRP) level was identified as a predictive factor for the onset of WON. We suggest the clinical importance of continuous monitoring at an early stage after diagnosis to identify the maximum CRP level.

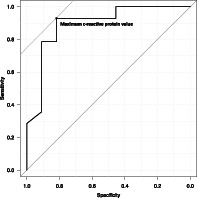

## Introduction

In 2011, a nationwide survey in Japan determined a pancreatitis incidence of 49/100 000 persons/year, and approximately 20% of these cases involve severe acute pancreatitis.^1^ Approximately 5–10% of patients with acute pancreatitis present with necrotic pancreatitis.^2^ Local complications of acute pancreatitis occurring within 4 weeks of onset are classified as acute necrotic collection (ANC) according to the 2012 revised Atlanta classification, while those occurring beyond 4 weeks are classified as walled‐off necrosis (WON).^3^ WON is reported to develop in 1–9% of patients with acute pancreatitis, among whom 40% exhibit symptoms requiring treatments such as infection and gastrointestinal obstruction.^4^, ^5^ Notably, severe acute pancreatitis with infectious pancreatic necrosis (including WON) is associated with high mortality rates of 24–32%, compared to rates of 0–11% for non‐necrotizing pancreatitis and 3.5–11% for non‐infectious pancreatic necrosis.^6^, ^7^, ^8^ Accordingly, the prediction of WON development is essential to enable the necessary rapid drainage of a suspected infection.^9^, ^10^

Although a review by Ibrahim A described some potential predictors of pancreatic necrosis, including the C‐reactive protein (CRP), lactate dehydrogenase (LDH), and procalcitonin levels in serum, the onset of WON and predictive factors remains uncertain.^11^ Contrast‐enhanced computed tomography (CT) is the gold standard for a diagnosis of ANC/WON but may not be feasible in patients with unstable vital signs, renal failure, or contrast media allergy. Therefore, it is clinically important to assess the risk of WON based on factors other than contrast‐enhanced CT findings.^12^, ^13^ Furthermore, a predictive marker of WON in the early stage of severe pancreatitis allows early identification of those patients who require transfer to a critical care medical center, treatment in an intensive care unit, and/or drainage therapy. In this study, therefore, we aimed to identify predictors of WON in a sample of patients with severe acute pancreatitis who were diagnosed at our hospital.

## Methods

### Setting

This was a single‐center retrospective, exploratory, cross‐sectional study.

### Study population

**Figure 1:**
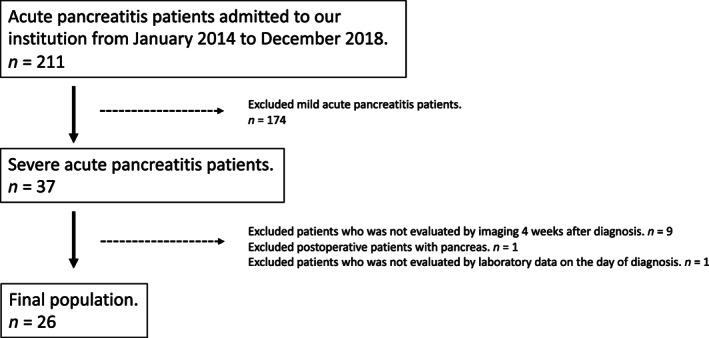
Patient selection flow chart.

For this study, 37 patients with severe acute pancreatitis were identified among 211 patients with acute pancreatitis who had been admitted to our hospital between January 2014 and December 2018. Among these 37 patients, we excluded 9 patients who did not undergo an imaging evaluation within 4 weeks after the onset of severe acute pancreatitis because they were transferred to another hospital or died after receiving a diagnosis at our hospital, 1 patient who underwent pancreatic surgery, and 1 patient for whom blood data on the day of diagnosis were unavailable. The remaining 26 patients with severe acute pancreatitis were included in the study (Fig. 1).

### Ethics and conflicts interest

This study was approved by the Ethical Review Board of Saitama Medical Center, Jichi Medical University (Approval No. S19‐087). Because this study is a retrospective study, posting an opt‐out document on our home page guaranteed the opportunity for the study subjects to refuse. This study has no conflicts of interest.

### Diagnostic and treatment protocol

We diagnosed and assessed the severity of acute pancreatitis using the 2008 revised Japanese Severity Score (JSS) developed by the Research Group on Intractable Pancreatic Diseases in the Research Program for Overcoming Intractable Diseases, which is funded by the Ministry of Health, Labour and Welfare in Japan (see the Supporting Information, which shows the 2008 revised JSS).^14^ In patients who could not undergo contrast‐enhanced CT because of renal failure, allergy, or other reasons, the extent of significant inflammatory changes was evaluated by plain CT, and unenhanced areas in the pancreatic parenchyma were defined as unevaluable.

The causes of acute pancreatitis were classified as alcohol‐induced, gallstone, post‐endoscopic retrograde cholangiopancreatography (ERCP), and others. We defined alcohol‐induced acute pancreatitis as the daily alcohol intake of four or more standard drinks (48 g/day), gallstone pancreatitis as the clear appearance of features of choledocholithiasis on imaging, and post‐ERCP pancreatitis as the emergence of new clinical signs of acute pancreatitis after an ERCP procedure, accompanied by increases in the serum levels of pancreatic enzymes at least three times above the upper limits of normal levels.^15^, ^16^ In this study, the compared items were factors associated with the diagnosis of acute pancreatitis, and the analysis included the values obtained at the time of diagnosis of severe acute pancreatitis (day 0). However, as the CRP level often peaks after a diagnosis, the maximum value within 7 days of diagnosis was also included for comparison.^14^, ^17^, ^18^

Basic treatment for severe acute pancreatitis was administered according to the 2010 and 2015 Japanese Guidelines for the Management of Acute Pancreatitis and comprised large‐volume fluid replacement, antibiotics, and protease inhibitors.^16^, ^19^ Patients with stable vital signs were admitted to the general ward. Those with unstable vital signs or whose vital signs became unstable after admission to the general ward were admitted to the intensive care unit (ICU), where systemic management was administered to improve the patient's condition before transfer to the general ward. The WON group (WG) included patients with an encapsulated collection with liquid and nonliquid density in the pancreatic parenchyma or around the pancreas observed on CT or magnetic resonance imaging (MRI) performed 4 or more weeks after the diagnosis of acute pancreatitis, while the non‐WON group (NWG) comprised patients without features of WON.^3^ Patients with asymptomatic WON were treated conservatively, while those with symptoms indicative of an infection, such as abdominal pain, fever, and an increased inflammatory response, were treated according to the step‐up approach.^20^ The first‐choice drainage procedures were endoscopic ultrasound (EUS)‐guided drainage for patients with stable vital signs and percutaneous drainage for those with unstable vital signs. Percutaneous drainage was added if no improvement was observed with EUS‐guided drainage alone. Endoscopic necrosectomy was performed for patients with a suspected collection of necrotic substances within the WON, as indicated by CT, and in whom the use of drainage alone was considered insufficient. Surgical treatment was performed for patients who responded inadequately to endoscopic necrosectomy.

### Statistical analysis

Categorical variables are expressed as numbers of subjects (percentages), while continuous variables are expressed as medians (ranges). The Fisher's exact test or the Mann–Whitney *U* test was used to compare the categorical and continuous variables within each group, respectively. Factors associated with the onset of WON were investigated using a multiple logistic regression analysis of variables that exhibited significant differences in a univariate analysis, including the presence of pancreatic necrosis on CT and the LDH and maximum CRP levels. The results of the multiple logistic regression analysis are expressed using odds ratios (ORs) and 95% confidence intervals (95% CIs), and the extracted factors were subjected to a receiver operating characteristic (ROC) curve analysis to determine the suitability for predicting the presence/absence of WON. Missing data are excluded from the analysis. A *P* value of <0.05 was considered to indicate a significant difference. All statistical analyses were performed using EZR software.^21^

## Results

WON occurred in 15 of the 26 patients included in this study (57.7%). The patients' characteristics are shown in Table 1. The patients had a median age of 67 years, and 65.4% (17/26) were male. The most common causes of acute pancreatitis were post‐ERCP in the WG and others in the NWG. Although the rate of complicating chronic pancreatitis was high in the NWG, it did not differ significantly from the rate in the WG. There were no marked differences in the rates of other complications between the groups.

**Table 1:** Patient characteristics

Parameters	All (*n* = 26)	WON group (*n* = 15)	Non‐WON group (*n* = 11)	*P* value
Age (years)	67 (44–86)	66 (44–81)	70 (47–86)	0.436
Male	17 (65.4%)	11 (73.3%)	6 (54.5%)	0.419
Etiology				0.938
Alcoholic	5 (19.2%)	3 (20.0%)	2 (18.2%)	
Biliary stones	3 (11.5%)	2 (13.3%)	1 (9.1%)	
Post‐ERCP	9 (34.6%)	6 (40.0%)	3 (27.3%)	
Others	9 (34.6%)	4 (26.7%)	5 (45.5%)	
History				
Chronic pancreatitis	7 (26.9%)	2 (13.3%)	5 (45.5%)	0.095
Liver cirrhosis	1 (3.8%)	0	1 (9.1%)	0.423
Heart disease	7 (26.9%)	5 (33.3%)	2 (18.2%)	0.658
Respiratory disease	0	0	0	—
Chronic kidney disease	2 (7.7%)	1 (6.7%)	1 (9.1%)	1
Diabetes	11 (42.3%)	7 (46.7%)	4 (36.4%)	0.701
BMI (kg/m^2^)	22.2 (15.5–34.0)	23.3 (19.6–34.0)	22.1 (15.5–27.9)	0.118
mBP (mmHg)	98 (74–142)	109 (81–142)	94 (74–123)	0.224
RR (/min)	18 (12–41)	21 (16–41)	17 (12–26)	0.010
BT (°C)	37.1 (35.8–38.4)	37.0 (36–38.4)	37.1 (35.8–38.4)	0.750
PR (/min)	86.5 (63–142)	90 (67–142)	79 (63–139)	0.100

Categorical data are shown as numbers, continuous data are shown as medians (range).

BMI, body mass index; BT, body temperature; ERCP, endoscopic retrograde chorangiopancreatography; mBP, mean blood pressure; PR, pulse rate; RR, respiratory rate; WON, walled‐off necrosis.

**Figure 2:**
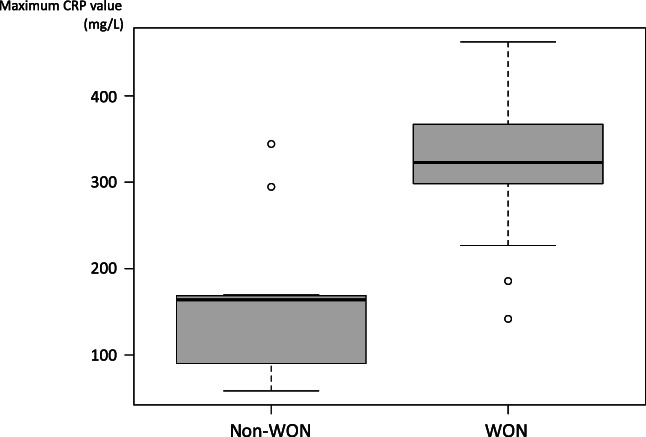
Box plot showing distribution the maximum C‐reactive protein (CRP) value in walled‐off necrosis (WON) group and non‐WON group.

As shown in Table 2, the median LDH levels in serum were 319 (207–867) U/L in the WG and 225 (177–492) U/L in the NWG (*P* = 0.049), while the corresponding median maximum CRP levels in serum were 322.7 (141.8‐462.4) and 163.8 (58.4‐344.2) mg/L, respectively (*P* = 0.001). There was significant overlap in the range of maximum CRP, so we show the box plot in Figure 2. Both inter‐group differences were significant. However, there were no significant differences in indicators of pancreatitis severity, such as the prognostic factors and CT grade. At the time of diagnosis of pancreatitis, 30.8% of the subjects (8/26) were unable to undergo a contrast‐enhanced CT assessment of the poorly perfused area of the pancreas (i.e., pancreatic necrosis) owing to decreased renal function. The proportion of patients unable to undergo contrast‐enhanced CT was higher in the WG (46.7%, 7/15) than in the NWG (9.1%, 1/11), although this trend was not significant (*P* = 0.084). Moreover, 50.0% of patients in the WG (4/8) and 20.0% in the NWG (2/10) were diagnosed with pancreatic necrosis by contrast‐enhanced CT, and this difference was not significant (*P* = 0.321).

**Table 2:** Comparison of laboratory data, SIRS score, and severity of pancreatitis between the WON group and non‐WON group

Laboratory data	All (*n* = 26)	WON group (*n* = 15)	Non‐WON group (*n* = 11)	*P*‐value
BUN (mg/dL)	16 (6–96)	18 (8–96)	16 (6–36)	0.222
Creatinine (mg/dL)	0.87 (0.22–5.66)	0.88 (0.45–5.66)	0.73 (0.22–2.35)	0.223
LDH (U/L)	273 (177–867)	319 (207–867)	225 (177–492)	0.049
Amylase (U/L)	966 (57–4176)	964 (230–4176)	1039 (57–3079)	0.403
White blood cell (/μL)	12 705 (1860–20 600)	12 610 (1860–20 600)	12 800 (2140–16 490)	0.760
Hemoglobin (g/dL)	13.8 (9.9–18.8)	14.3 (11.9–18.8)	12.6 (9.9–16.2)	0.113
Hematocrit (%)	41.5 (30.1–54.7)	43.3 (33.5–54.7)	37.8 (30.1–47)	0.148
Platelet (×10^4^/μL)	20.4 (2.4–38.5)	20.5 (2.4–38.5)	19.7 (6.2–30)	0.378
Revised calcium (mg/dL)	8.9 (6.3–9.9)	8.95 (6.3–9.5)	8.85 (8.4–9.9)	0.509
CRP (mg/L)	31.5 (0.3–428.6)	42.8 (0.3–428.6)	13.2 (0.4–335.8)	0.121
Maximum CRP (mg/L)	294.6 (58.4–462.4)	322.7 (141.8–462.4)	163.8 (58.4–344.2)	0.001
PT (%)	88.8 (52.8–100)	76.7 (54–100)	90 (52.8–100)	0.536
pH	7.425 (7.303–7.613)	7.435 (7.392–7.613)	7.402 (7.303–7.483)	0.224
PaO_2_ (Torr)	77.4 (56.8–110.5)	91.1 (56.8–110.5)	74.7 (65.5–95.2)	0.902
Lactate (mg/dL)	23 (7.3–45.6)	23.5 (12.2–39)	18.7 (7.3–45.6)	0.628
SIRS score				0.614
≤2	22 (84.6%)	12 (80.0%)	10 (90.9)	
≥3	4 (15.4)	3 (20.0%)	1 (9.1%)	
Severity of pancreatitis				
Prognostic factor				0.197
≤2	20 (77%)	10 (66.7%)	10 (90.9%)	
≥3	6 (23%)	5 (33.3%)	1 (9.1%)	
CE‐CT criteria				
Extension of extrapancreatic inflammatory changes				1
1	1 (4%)	1 (6.7%)	0	
2	25 (96%)	14 (93.3%)	11 (100%)	
Unenhanced area in the pancreatic parenchyma (n = 18)				0.321
0	12 (66.7%)	4 (50.0%)	8 (80.0%)	
1	6 (33.3%)	4 (50.0%)	2 (20.0%)	
Unevaluable	8 (30.8%)	7 (46.7%)	1 (9.1%)	0.084
CE‐CT grade				0.492
1	1 (4%)	1 (6.7%)	0	
2	23 (88%)	12 (80.0%)	11 (100%)	
3	2 (8%)	2 (13.3%)	0	

Categorical data are shown as numbers, and continuous data are shown as medians (range).

BUN, blood urea nitrogen; CE‐CT, contrast enhanced computed tomography; LDH, lactate dehydrogenase; PaO_2_, partial pressure of oxygen in arterial blood; PT, prothrombin time; SIRS, systemic inflammatory response syndrome; WON, walled‐off necrosis.

**Figure 3:**
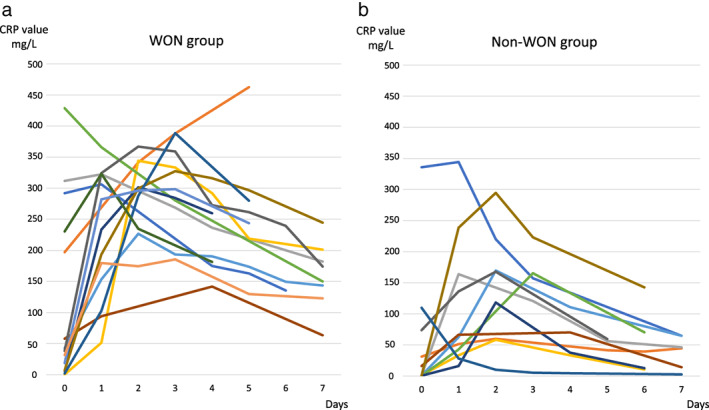
Change over time of C‐reactive protein (CRP) value in walled‐off necrosis (WON) group and non‐WON group. “a” to “y” indicate cases. The time until the CRP value reached the maximum value (median, range) was 2 (0–5) days, and 88% (22/25) was within 3 days. (a) WON group: 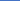, a; 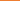, b; 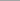, c; 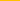, d; 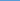, e; 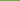, f; 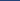, g; 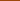, h; 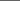, i; 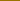, j; 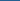, k; 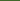, l; 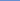, m; 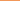, n. (b) Non‐WON group: 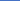, o; 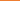, p; 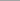, q; 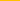, r; 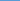, s; 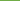, t; 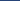, u; 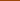, v; 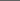, w; 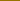, x; 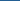, y.

**Figure 4:**
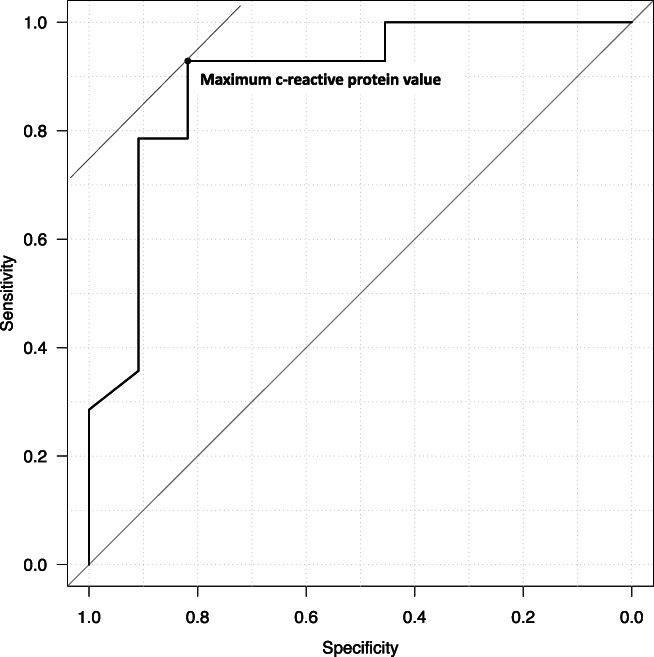
Receiver operating characteristics curve for predicting walled‐off necrosis occurrence. AUC, area under the curve. Cutoff, 185.5; sensitivity, 0.929; specificity, 0.818; positive predictive value, 0.867; negative predictive value, 0.900; AUC, 0.893.

The multivariate logistic regression analysis of potential predictors of pancreatic necrosis identified the maximum CRP level as a risk factor for WON (OR: 1.20, 95% CI: 1.05–1.37, Table 3). Figure 3 presents changes in the CRP levels. In one patient in the WG, the maximum CRP level could not be determined because this parameter was measured only once before day 7. Among the remaining patients, the median time to reach the maximum CRP level was 2 (0–5) days, and 88% of patients (22/25) reached this level within 3 days. We then used a previously reported CRP level of 300 mg/L as a cut‐off for distinguishing infectious from non‐infectious pancreatic necrosis and determined that 11 patients, including 10 of 14 (71.4%) in the WG and 1 of 11 (9.1%) in the NWG, had a maximum CRP level that exceeded 300 mg/L.^17^ Of these 11 patients, 81.8% (9/11) had a CRP level greater than 300 mg/L within 2 days. Our ROC analysis identified a maximum CRP cutoff value for the onset of WON of 185.5 mg/L, with a sensitivity of 0.929, specificity of 0.818, positive predictive value of 0.867, negative predictive value 0.900, and area under the curve (AUC) of 0.893 (Fig. 4).

**Table 3:** Multivariate analysis of WON occurrence

	Odds ratio	95% confidence interval	*P* value
Pancreatic necrosis	1.73	0.59–50.4	0.751
LDH	1.01	0.996–1.02	0.204
Maximum CRP	1.20	1.05–1.37	0.0076

CRP, c‐reactive protein; LDH, lactate dehydrogenase; WON, walled‐off necrosis.

As shown in Table 4, there were no significant differences between the two groups in terms of treatment approaches. Drainage was performed in eight patients (53.3%) in the WG, including EUS‐guided transgastric drainage in five patients, percutaneous drainage in one, EUS‐guided transgastric drainage plus percutaneous drainage in one, and percutaneous drainage plus surgical drainage (e.g., open necrosectomy) in one patient. Three patients in the WG group (11.5%) died during admission; however, there was no significant difference in this variable between the groups. The causes of death during admission included severe acute pancreatitis associated with WON infection in two cases, and underlying disease (gallbladder cancer) in one case.

**Table 4:** Treatments and mortality of severe acute pancreatitis

	All (*n* = 26)	WON group (*n* = 15)	Non‐WON group (*n* = 11)	*P* value
Infusion volume (〜24 h)	3916 (2350–6893)	3800 (2350–6893)	3916 (2400–5000)	0.784
Antibiotics				0.074
MEPM	12 (48.0%)	8 (57.1%)	4 (36.4%)	
SBT/CPZ	4 (16.0%)	3 (21.4%)	1 (9.1%)	
SBT/ABPC	1 (4.0%)	1 (7.1%)	0	
IPM/CS	2 (8.0%)	0	2 (18.2%)	
TAZ/PIPC	3 (12.0%)	0	3 (27.3%)	
CPZ	1 (4.0%)	1 (7.1%)	0	
CEZ	1 (4.0%)	0	1 (9.1%)	
PIPC	1 (4.0%)	1 (7.1%)	0	
Not used	0	0	0	
Protease inhibitors				0.148
FUT	16 (61.5%)	7 (46.7%)	9 (81.8%)	
FOY	3 (11.5%)	3 (20.0%)	0	
FOY + UTI	1 (3.8%)	0	1 (9.1%)	
FUT + UTI	3 (11.5%)	2 (13.3%)	1 (9.1%)	
Not used	3 (11.5%)	3 (20.0%)	0	
Death during hospitalization	3 (11.5%)	3 (20.0%)	0	0.238
Drainage		8 (53.3%)		
EUS		5 (62.5%)		
Percutaneous		1 (12.5%)		
Percutaneous + EUS		1 (12.5%)		
Percutaneous + surgical necrosectomy	1 (12.5%)			

Categorical data are shown as numbers, and continuous data are shown as medians (range).

ABPC, ampicillin; CEZ, cefazolin; CPZ, cefoperazone; CS, cilastatin; EUS, endoscopic ultrasound; FOY, gabexate mesylate; FUT, nafamostat mesylate; IPM, imipenem; MEPM, meropenem; PIPC, pepiracillin; SBT, sulbactam; TAZ, tazobactam; UTI, ulinastatin; WON, walled‐off necrosis.

## Discussion

Our single‐center retrospective, exploratory, cross‐sectional study yielded three main findings. First, the maximum CRP level measured between the time of diagnosis of severe acute pancreatitis and day 7 postdiagnosis was identified as a statistically significant factor related to the onset of WON. Second, the CRP level peaked within 3 days and reached 300 mg/L within 2 days in most cases of the WON group. Third, CT findings of pancreatic necrosis could be obtained only in a small number of patients in the WG, as many patients were unable to undergo contrast‐enhanced CT at the time of diagnosis. Continuous monitoring of CRP levels during the early stage of severe acute pancreatitis is important for predicting the development of WON, especially when contrast‐enhanced CT is not available.

The CRP level is a prognostic factor for severe acute pancreatitis and has been identified as a predictive factor for both pancreatic necrosis and infective pancreatic cysts.^11^, ^17^, ^22^ In this study, patients in the WG had a median maximum CRP level of 322.7 mg/L, and 88% reached a peak value within 3 days. Moreover, the ROC analysis identified a maximum CRP cutoff of 185.5 mg/L with an AUC of 0.893, suggesting that the maximum CRP level may be useful as a predictor of WON. The CRP is generally thought to peak within 48 h after the onset of an infection or inflammatory disease, and therefore, patients with severe acute pancreatitis should be evaluated at the time of diagnosis and 48 h after onset.^16^, ^18^ However, the time interval from the onset to the diagnosis of severe acute pancreatitis at a medical institution often differs between patients. In this study, we observed variations in the time required to reach a peak CRP level, as the maximum CRP level was reached prior to diagnosis in some patients, but was not reached until several days after onset in other patients. Therefore, in actual clinical practice, the length of time from the true onset of severe acute pancreatitis to a hospital visit mostly depends on the patient. One previous report indicated that the CRP level rarely reaches 300 mg/L within 3–4 days in patients with severe acute pancreatitis.^17^ However, in this study, 88% of patients reached a maximum CRP level within 3 days and 81.8% reached a CRP of 300 mg/L within 2 days in the WG despite variations in the intervals between the time of onset and the hospital visit. Therefore, caution should be taken to changes in CRP levels during the early course of the disease.

Interestingly, in our WG, only 50.0% (4/8) of patients who underwent contrast‐enhanced CT at the time of diagnosis of severe acute pancreatitis were eventually diagnosed with pancreatic necrosis, whereas 46.7% (7/15) could not be evaluated via CT because of poor renal function. In other words, when we combined patients who were not diagnosed with pancreatic necrosis on contrast‐enhanced CT and those who could not undergo this type of imaging analysis, 73.3% (11/15) of patients in the WG were not diagnosed with pancreatic necrosis. Rather, these patients were diagnosed as having severe acute pancreatitis according to the JSS, based on the presence of fluid collection around the pancreas that extended beyond the inferior pole of the kidney. An evaluation of pancreatic necrosis by contrast‐enhanced CT is extremely important, as this analysis has been reported to be correlated with mortality. Conversely, the risk of developing WON over time must be considered in patients without obvious necrosis on imaging or those who lacked contrast‐enhanced CT imaging data. Therefore, it is considered highly significant to monitor the CRP levels, in addition to the CT findings.^23^, ^24^

CRP testing is clinically feasible because it can be performed in a simple, minimally invasive manner. The use of the maximum CRP level as an index allows, for example, an infection assessment and preliminary discussion of the timing of CT re‐imaging and drainage. Patients with symptomatic WON require early drainage, and effective low‐risk treatments such as endoscopic drainage and endoscopic necrosectomy are recommended.^10^, ^25^, ^26^, ^27^, ^28^ However, the feasibility of these procedures is often limited by the institution, and prompt transfer to another hospital should be considered in such cases. Taken together, these findings indicate that the close monitoring of CRP for at least 1 week after the diagnosis of severe acute pancreatitis, with the aim of identifying a clear peak, is considered clinically essential for controlling WON.

This study had several limitations. First, this was a single‐center retrospective study with a small sample size of 26. Second, we used diagnostic criteria for severe acute pancreatitis that were specific to Japan and may be slightly different from those used internationally. However, Japan's JSS is simple and was reported to yield a higher AUC than various international diagnostic criteria (e.g., Ranson score, Bedside Index of Severity in Acute Pancreatitis score, and Acute Physiology and Chronic Health Evaluation II score).^29^, ^30^, ^31^ Therefore, the application of the JSS was unlikely to pose major clinical problems. Third, this study included a high percentage of cases of post‐ERCP pancreatitis, which is iatrogenic and may have a different mechanism from that of usual acute pancreatitis.^32^ Combined investigation of different etiologies may be questionable. Our findings should be interpreted in consideration of these limitations.

## Conclusions

In this investigation, we identified the maximum CRP level as a predictive factor for the onset of WON. Specifically, a high percentage of patients who developed WON exhibited elevated CRP levels within 2 days of diagnosis. Our findings suggest the importance of continuous monitoring of CRP levels during the early stage of severe acute pancreatitis to predict the development of WON for a better outcome.

## Supporting information


**Appendix** **S1.** Supporting information.
