# Supplementary material for: C‐reactive protein predicts the development of walled‐off necrosis in patients with severe acute pancreatitis
Source: JGH Open. 2021 Jun 29;5(8):907–14. doi: 10.1002/jgh3.12605 (PMC8341195; doi:10.1002/jgh3.12605)
Supplement: Supplementary file 1 — Appendix S1. Supporting information. [file JGH3-5-907-s001.pdf]

Supporting information

The severity criteria for acute pancreatitis developed by the Japanese Ministry of Health, Labor and Welfare in 2008

Prognostic factors (one point for each positive factor)

1. Base excess $\leq$ -3 mEq/L or shock (systolic blood pressure < 80 mmHg)
2. $PaO_2 \leq 60$ mmHg or respiratory failure (needing respirator)
3. BUN $\geq$ 40 mg/dL (or Cr $\geq$ 2 mg/dL) or oliguria (<400 ml/day even after fluid therapy)
4. Elevation of LDH twice or more than upper normal limit
5. Platelet count $\leq$ 100,000/µL
6. Serum calcium $\leq$ 7.5 mg/dL
7. CRP $\geq$ 15 mg/dL
8. Meeting 3 or more SIRS criteria (body temperature > 38°C or <36°C, heart rate > 90/min, respiratory rate > 20/min or $PaCO_2$ < 32 torr, WBC > 12,000/µL or < 4000/µL or > 10% immature leukocyte)
9. Age $\geq$ 70 years

Contrast-enhanced computed tomography criteria

1. Extrapancreatic progression of inflammation

Anterior pararenal space	0 point
Root of mesocolon	1 point
Beyond lower of kidney	2 points

2. Hypo-enhanced region of pancreas

The pancreas is divided into three segments; head, body and tail

Localized within each segment or only surrounding the pancreas	0 point
Covers 2 segments	1 point
Occupies entire $\geq$2 segments	2 points

1+2=Total score

Total score= 0 or 1	Grade 1
Total score= 2	Grade 2
Total score= $\geq$3	Grade 3

Severe AP is defined as $\geq$3 positive prognostic factors or revealing CT grade 2 or more.
